# ERPs Reveal the Time-Course of Aberrant Visual-Phonological Binding in Developmental Dyslexia

**DOI:** 10.3389/fnhum.2016.00071

**Published:** 2016-03-01

**Authors:** Manon W. Jones, Jan-Rouke Kuipers, Guillaume Thierry

**Affiliations:** ^1^School of Psychology, Bangor UniversityBangor, UK; ^2^Department of Psychology, University of StirlingStirling, UK

**Keywords:** dyslexia, letter identification, binding, ERPs (event related potentials), mismatch negativity (MMN), lateralized readiness potential

## Abstract

New evidence is accumulating for a deficit in binding visual-orthographic information with the corresponding phonological code in developmental dyslexia. Here, we identify the mechanisms underpinning this deficit using event-related brain potentials (ERPs) in dyslexic and control adult readers performing a letter-matching task. In each trial, a printed letter was presented synchronously with an auditory letter name. Incongruent (mismatched), frequent trials were interleaved with congruent (matched) infrequent target pairs, which participants were asked to report by pressing a button. In critical trials, incongruent letter pairs were mismatched but confusable in terms of their visual or phonological features. Typical readers showed early detection of deviant trials, indicated by larger modulation in the range of the phonological mismatch negativity (PMN) compared with standard trials. This was followed by stronger modulation of the P3b wave for visually confusable deviants and an increased lateralized readiness potential (LRP) for phonological deviants, compared with standards. In contrast, dyslexic readers showed reduced sensitivity to deviancy in the PMN range. Responses to deviants in the P3b range indicated normal letter recognition processes, but the LRP calculation revealed a specific impairment for visual-orthographic information during response selection in dyslexia. In a follow-up experiment using an analogous non-lexical task in the same participants, we found no reading-group differences, indicating a degree of specificity to over-learnt visual-phonological binding. Our findings indicate early insensitivity to visual-phonological binding in developmental dyslexia, coupled with difficulty selecting the correct orthographic code.

## Introduction

Developmental dyslexia involves impaired reading and writing, in the absence of a more general cognitive impairment (Lyon et al., 2003). Causes of dyslexia remain heavily debated (c.f., Gori and Facoetti, 2014; Norton et al., 2015), but accumulating evidence indicates that difficulty in forming associations between visual-orthographic and corresponding phonological codes is a reliable indicator, which persists into adulthood (Price and Devlin, 2003; Devlin et al., 2006; Shaywitz and Shaywitz, 2008; Hellyer et al., 2011; Kherif et al., 2011; Schurz et al., 2015). In this study, we examine the temporal dynamics of learned visual-phonological connections using electrophysiological methods. Specifically, we compared the ability of adult typical and dyslexic readers to recognize and respond to highly practiced associations between a printed letter and its name.

Models of reading consider links between visual-orthographic inputs and phonological outputs as created over multiple exposures to orthographic-phonological correspondences (e.g., Seidenberg and McClelland, 1989; Harm and Seidenberg, 1999; Manis et al., 1999). With practice, these links enable fast, automatic access to phonological codes, promoting fluent reading (LaBerge and Samuels, 1974; Logan, 1997). However, readers with dyslexia remain unable to fully automatize this process. Even highly compensated adult dyslexic readers are significantly slower to name sequences of letters in tasks such as rapid automatized naming (RAN), compared with their typically developed peers (e.g., Lefly and Pennington, 1991; Bruck, 1998; Shaywitz and Shaywitz, 2008; for reviews, see Wolf and Bowers, 1999; Kirby et al., 2010; Norton and Wolf, 2012). Recent evidence shows that dyslexic readers are maximally impaired in serial naming tasks, for which multiple letter representations compete for output (Jones et al., 2008, 2013a; Yan et al., 2013). However, *individual* letter presentation also incurs a significant—albeit smaller—naming speed cost (Bowers and Swanson, 1991; Castel et al., 2008; Jones et al., 2009). Although the cause of this fundamental difficulty remains largely underspecified, it appears to be key to understanding visual-phonological mapping.

In neurocognitive terms, establishing links between visual-orthographic stimuli and phonological output must involve a cross-modal binding mechanism. Letter naming recruits mid-fusiform areas, which play a role in identifying objects and words, forming close connections with language regions responsible for name retrieval and semantic processing (Dehaene et al., 2005; Price et al., 2006; Lervåg and Hulme, 2009). The learning process itself must also incur significant working memory demands, with a particular role for executive processes in maintaining cross-modal connections in the episodic buffer (c.f., Jones et al., 2013b). Paired associate learning (PAL) tasks—in which novel connections are created between a visual stimulus and a verbal response—attest to the unique role of cross-modal demands in reading. PAL response accuracy—following a mere five or so learning trials—predicts word and nonword reading accuracy, as well as reading speed (Hulme et al., 2007; Warmington and Hulme, 2012), but this predictive relationship is found only for visual-verbal (and not visual-visual) versions of the task (Messbauer and de Jong, 2003). Dyslexic readers are also consistently poorer at PAL visual-verbal tasks compared with typical readers (Vellutino et al., 1995; Wimmer et al., 1998; Messbauer and de Jong, 2003; Jones et al., 2010).

Event related potentials (ERPs) provide a high-resolution timeline of participants’ neurocognitive responses, and are therefore ideally suited to explore sources of deviancy between dyslexic and typical readers before the observation of a response. Froyen and colleagues have used ERPs to examine automaticity in letter naming to good effect. In these studies, typical readers showed larger mismatch negativity (MMN) in response to printed letters presented with mismatched auditory letter sounds. A commensurate effect was not found in dyslexic readers, suggesting a deficit in pre-attentive, automatic letter-speech sound processing (Froyen et al., 2009, 2011; see also Mittag et al., 2013). However, these findings remain silent on the possible causes of aberrant connection between the visual and the phonological code.

In this study, we used ERPs to examine whether difficulties with letter processing in dyslexia primarily relate to visual-orthographic or phonological processing (e.g., Elbro, 1996; Brady, 1997; Metsala, 1997; Adlard and Hazan, 1998; Snowling, 2001; Morais, 2003; Boada and Pennington, 2006; Facoetti et al., 2006, 2008; Vidyasagar and Pammer, 2010). We thus manipulated visual-orthographic and phonological similarity (and therefore confusability) in the context of letter identification. In Experiment 1, two groups of typical and dyslexic readers viewed individual letters synchronously with auditory presentation of a letter name. On most trials, letter forms and names were incongruent (e.g., v presented with /m/), and participants were required to respond with a button press to the small proportion of trials in which letter forms and names were congruent (e.g., p presented with /p/). Crucially, an equally small proportion of deviant trials comprised letter forms and name pairs that were incongruent, but were very similar to congruent trials, either in based on their visual or their phonological properties (e.g., q − /p/ and q − /k/ respectively). Thus, deviant trials elicited confusion as regards letter-name matching (see Jones et al., 2008, 2013a, for a similar letter-confusability manipulation). In order to test whether findings from the letter-matching task in Experiment 1 were language-specific, the same participants performed an equivalent task with non-alphabetic visual stimuli (geometric shapes) and nonverbal sounds (tones) in Experiment 2.

Of critical interest in both experiments was the degree to which visual or phonological deviant trials modulated electrophysiological responses in typical and dyslexic readers. For letter stimuli (Experiment 1), the phonological mismatch negativity (PMN) is a modulation in the N2 range known to index interactions between lexical processing and phonological expectations. PMN differences typically occur between 250–350 ms post-stimulus, with larger negative-going amplitudes elicited by phonologically unexpected—or “deviant”—stimuli (Connolly and Phillips, 1994; Hagoort and Brown, 2000; Diaz and Swaab, 2007). For non-linguistic stimuli (Experiment 2), an analog of the PMN in the nonverbal domain, the MMN, indexes deviancy elicited by visually or auditorily unexpected stimuli, between 150–250 ms post stimulus onset (c.f., Näätänen, 1992).

The P3b measured in both experiments is typically elicited by an unexpected or improbably event. It is associated with explicit attentional engagement, and is closely yoked to the behavioral response (Polich, 2007). In this study, a larger P3b was expected in response to deviants, reflecting the close similarity of visually and auditorily presented stimuli as compared to stimuli presented in the standard trials (Polich, 2007). We also calculated the lateralized readiness potential (LRP), which reflects late-stage motor response preparation, in this study related to preparation of a button press response executed by the right index finger. We used the mean amplitudes of the PMN/MMN, P3b, and LRP to measure early detection, recognition, and response preparation of deviant stimuli as compared to standard stimuli. A non-verbal response moreover ensured that any difficulties shown in the dyslexic group at this stage could be identified as a problem in selection *per se*, rather than impairment relating to producing a voiced response (e.g., initiating the articulators: c.f., Fawcett and Nicolson, 2002).

## Experiment 1: Method

### Participants

Two groups of 20 “typical” and “dyslexic” readers were recruited; all native British-English speaking students. Data from two participants from each group were omitted from the analyses owing to <20 artifact free trials per condition. The final set therefore comprised 18 “dyslexic” participants (age: *M* = 21.15, *SD* = 2.54; gender: 12 females), all of whom had been formally assessed by an Educational Psychologist during primary or secondary education and a further 18 participants in the “typical” group (age: *M* = 20.76, *SD* = 2.63; gender: 9 females), all of whom reported no difficulties associated with literacy. All participants had normal or corrected vision and reported no other problems (e.g., hearing loss, specific language impairment, Attention deficit hyperactivity disorder (ADHD) etc.). The study was approved by the Ethics Committee, Bangor University and participants received payment for participation.

### Literacy and General Cognitive Ability

Allocation of participants to reading groups was validated via a short battery of tests. Word reading efficiency and phonemic decoding efficiency subscales of the *Test of Word Reading Efficiency* (TOWRE, Torgesen et al., 1999) were used, in addition to vocabulary (verbal) and matrix reasoning (nonverbal) indices of intelligence quotient (IQ) from the *Wechsler Abbreviated Scale of Intelligence* (WASI, Wechsler, 1999). Digit and letters version of the RAN task were obtained from the *Comprehensive Test of Phonological Processing* (CTOPP; Wagner et al., 1999).

### Stimuli, Design and Procedure

On each trial, a single letter was presented at center screen, subtending a visual angle of 1 degree (participants sat at a distance of 75 cm from the monitor). A letter name was presented simultaneously via loudspeakers (44 kHz). Participants were asked to execute a button press, using their right index finger, in response to target stimuli (see below). Stimulus presentation persisted for 1000 ms or until the participant responded (1500 ms ISI).

*Target* trials (*n* = 72 in total)—which required a button press response—presented congruent letter/letter name pairs.

**Figure 1:**
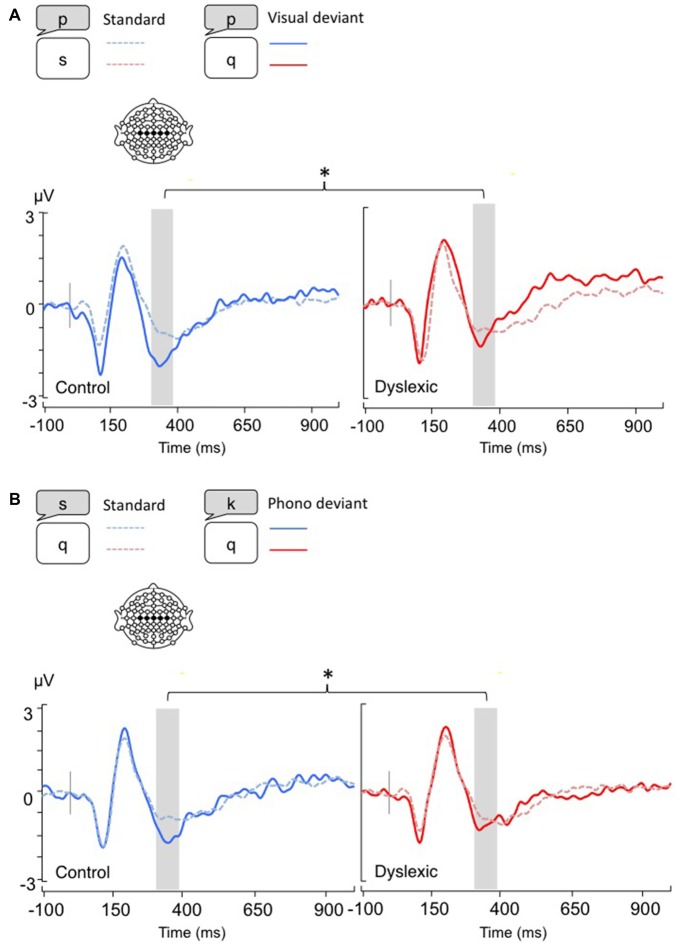
**Linear derivations over the electrodes where the phonological mismatch negativity (PMN) was maximal for typical and dyslexic readers in correct trials (Gray bar: analysis window). (A)** Visual-orthographic deviants; **(B)** Phonological deviants. *p < 0.05.

**Figure 2:**
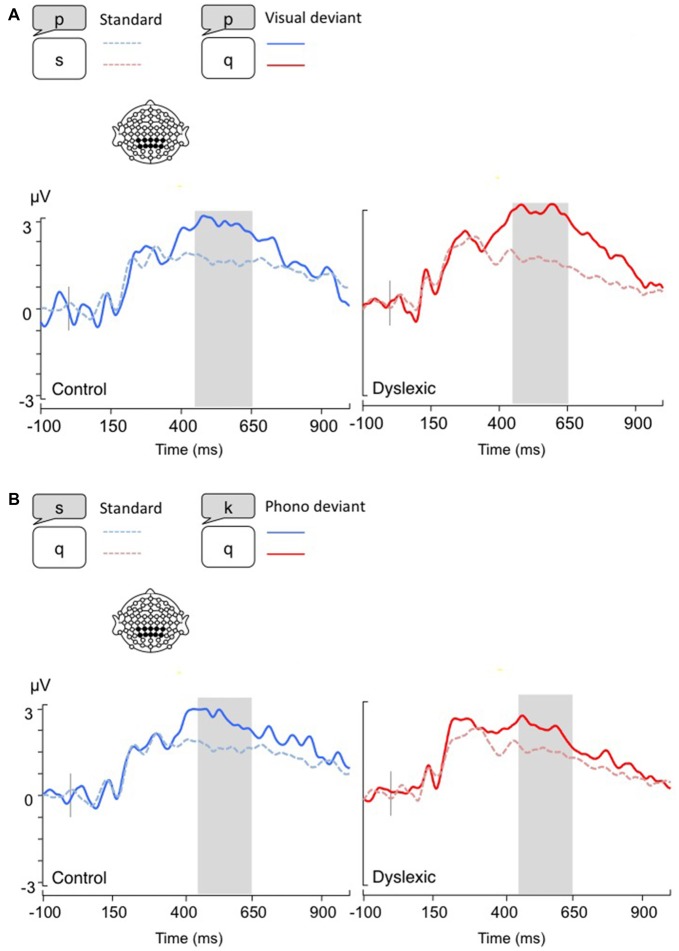
**Linear derivations over the electrodes where the P3b was maximal for typical and dyslexic readers’ responses in correct trials (Gray bar: analysis window). (A)** Visual-orthographic deviants; **(B)** Phonological deviants.

**Figure 3:**
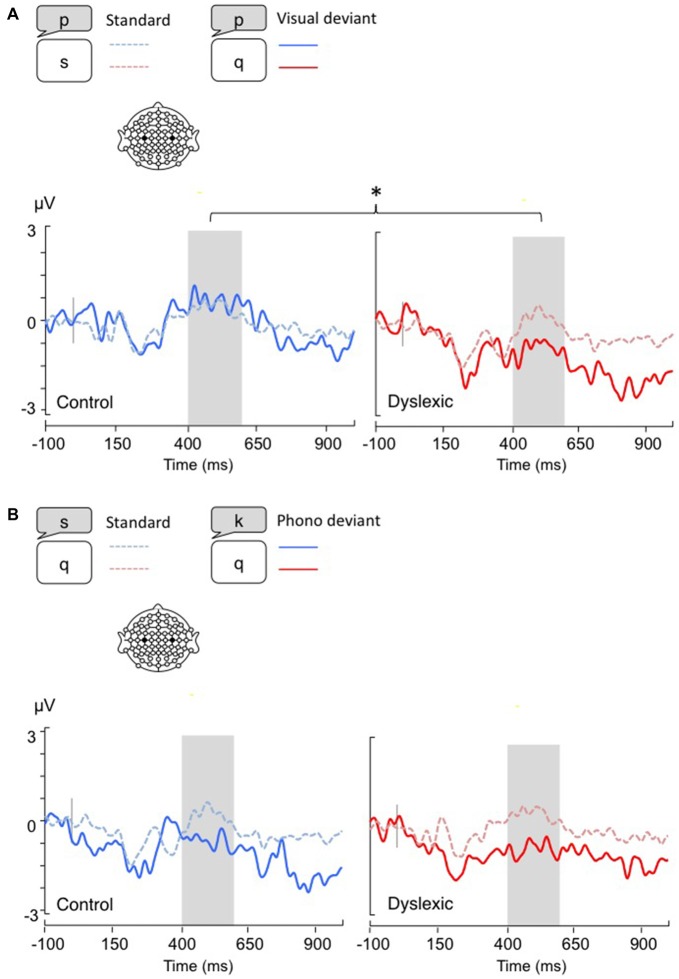
**Lateralized readiness potentials (LRPs), calculated by subtracting C4 amplitudes from C3.** Gray bar: analysis window. **(A)** Visual-orthographic deviants; **(B)** Phonological deviants. *p < 0.05.

*Deviant* trials (*n* = 72 in total) were incongruent and did not therefore require a button press response. However, pairs were strategically mismatched for visual or phonological similarity. Visual decoys (*n* = 36) comprised the pairs: s − /z/, d − /b/, p − /q/, z − /s/, b − /d/, q − /p/, s − /z/. Phonological decoys (*n* = 36) comprised the pairs: k − /q/, s − /x/, g − /j/, q − /k/, x − /s/, j − /g/ (see Figures 1–3).

*Standard* trials (*n* = 360 in total) comprised incongruent letter-name pairs (e.g., the letter “v” paired with the letter name /m/). These trials comprised fully rotated pairings of the letters c, f, h, l, m, n, r, t, v, y, with the exception of certain pairs, which were removed on the basis of overlapping visual and/or phonological features (e.g., m − n).

A subset of *super*-*standard* trials (*n* = 144/360) presented immediately prior to either a target or deviant trial comprised incongruent letter/letter-names, selected from the same subset of letters that were presented in target/deviant trials (e.g., s − /g/). Identical letter items between conditions (varying only in congruency of letter pairs) minimized error variance in the statistical comparison of conditions. Only data from these super-standard trials were therefore included in the analyses.

The number of standards between each target/deviant trial varied between two, three and four trials, such that correct trials constituted 16% of all trials presented in the experiment. Participants were given a short practice session before the experiment commenced, and a break on every 72nd trial.

### ERP Recording

Electrophysiological data was recorded at a rate of 1 kHz from 64 Ag/AgCl electrodes referenced to Cz and positioned according to the extended 10–20 convention. Electroencephalogram (EEG) activity was filtered online with a band-pass filter between 0.1 and 200 Hz and offline with a low-pass zero-phase shift digital filter that was set at 30 Hz (48 db/Oct). Eye blink artifacts in the EEG data were mathematically corrected using Neuroscan Software. The algorithm is derived from the method put forward by Gratton et al. (1983). Epochs were selected ranging from −100 to 1000 ms relative to the onset of the stimulus compound (visual and auditory letters). Epochs with activity exceeding ±75 μV at any electrode site were automatically discarded. There was an average of 32 trials per deviant condition in the typically developed participants and 34 trials on average for the dyslexic participants. Baseline correction was performed over the pre-stimulus interval, and individual averages were digitally re-referenced to the global average reference. Behavioral data were collected simultaneously to the ERP data.

### ERP Data Analysis

Mean PMN amplitude (Connolly and Phillips, 1994; Hagoort and Brown, 2000) was calculated over five central electrodes (C1, C2, C3, C4, Cz) across a 60 ms time-window around the maximal modulation for correct trials (320–380 ms).^1^ Mean P3b amplitude was calculated over ten parietal electrodes typically associated with the P3b (CP1, CP2, CPz, CP3, CP4, P1, P2, Pz, P3, P4; Polich, 2007) across a 200 ms time-window (450–650 ms).

In order to obtain an index of response selection, manual response preparation in both groups was measured via the LRP, which involved subtracting activity recorded at C4 from C3 (e.g., Coles, 1989). Participants were instructed to respond with the right index finger only.

Mean ERP amplitudes (PMN, P3b and LRP) were subjected to mixed-model analysis of variances (ANOVAs) comprising the within-subject factor Deviancy (standard letter vs. deviant letter) and the between-subjects factor Group (typical vs. dyslexic readers). Separate analyses were conducted for visual-orthographic and phonological deviants, as per our previous studies (e.g., Jones et al., 2008, 2013a, 2015) owing to the difficulty in equating feature overlap across visual and phonological domains.

ERP analyses were conducted only on correctly judged deviants (i.e., the participant did not execute a button press response). Correct trials were excluded from all analyses, since—unlike standard and deviant trials—they required an explicit button press response.

### Results

Background cognitive and literacy measures validated group differences on key measures, summarized in Table 1. Consistent with the diagnosis, the group with dyslexia read significantly fewer words and nonwords accurately, showed longer reading times and longer naming times in RAN. Each member of the dyslexic group obtained a score that was 1.5 SD below the control average on at least one of the key literacy measures (word/non-word reading, RAN). None were therefore excluded from the analysis on the basis of their literacy scores. Crucially, IQ performance was similar across groups.

**Table 1:** **Reading scores on cognitive and literacy tests**.

	Mean (SD)	
	Typical *N* = 18	Dyslexic *N* = 18	*t*	*Cohen’s d*	
Word reading (Acc)^a^	0.99 (0.00)	0.98 (0.01)	3.50**	0.14
Word reading (RT)^b^	52.83 (8.93)	79.94 (14.96)	−6.60***	2.20
Nonword reading (Acc)^a^	0.95 (0.04)	0.88 (0.07)	3.65**	1.22
Word reading (RT)^b^	53.38 (16.41)	78.77 (29.60)	−3.18*	1.06
RAN (RT)^b^	12.61 (1.87)	17.69 (2.88)	−6.25***	2.09
Verbal-IQ^c^	66.78 (5.63)	65.83 (6.61)	−0.46	0.15
Nonverbal-IQ^c^	54.89 (6.25)	54.06 (6.07)	−0.38	0.13

Note: ^a^Proportions; ^b^Time in seconds; ^c^T-scores. *p < 0.05; **p < 0.01; ***p < 0.001.

### Behavioral Results

Group differences in accuracy and RT were examined using linear mixed models implemented in R using the lme4 package (version 1.1–7; Bates et al., 2014). Both reading groups showed the same degree of accuracy in detecting correct trials (Typical *M* = 89%; Dyslexic *M* = 88%; *b* = −1.11, *z* = −0.52, *p* = 0.64), but dyslexic readers were faster to do so on average (Typical *M* = 694 ms; Dyslexic *M* = 567 ms; *b* = −126, *t* = −10.39, *p* < 0.001). Dyslexic readers were marginally more likely to make an erroneous button press in response to visual-orthographic deviant trials compared with typical readers, (Typical *M* = 92%; Dyslexic *M* = 88%; *b* = −0.59, *z* = −1.95, *p* = 0.05), but both reading groups showed the same degree of accuracy on phonologically deviant trials (Typical *M* = 98%; Dyslexic *M* = 98%; *b* = 0.20, *z* = 0.74, *p* = 0.77).

### ERP Results

A series of *t*-tests showed no significant group differences on the number of trials per condition included in the analyses (Standards: Typical readers *M* = 125, *SD* = 14; Dyslexic readers *M* = 128, *SD* = 8; *t* = 0.37; Visual-orthographic deviants: Typical readers *M* = 30, *SD* = 4; Dyslexic readers *M* = 31, *SD* = 3; *t* = 0.47; Phonological deviants: Typical readers *M* = 30, *SD* = 4; Dyslexic readers *M* = 32, *SD* = 2; *t* = 0.21).

*The PMN analysis (320–380 ms)* yielded no main effects (*p*s > 0.05), but a significant Deviancy * Group interaction emerged for both deviancy types: typical readers showed increased PMN amplitudes in relation to deviant trials compared with standard trials, but a similar modulation was not apparent in dyslexic readers (Visual-orthographic: *F*_(1,34)_ = 4.7, *p* < 0.05, *η* = 0.16; Phonological: *F*_(1,34)_ = 6.1, *p* < 0.05, *η* = 0.15; see Figure 1).

*The P3b analysis (450–650 ms)* showed a significant main effect of Deviancy, such that deviant trials elicited significantly larger P3b responses than standard trials (Visual-orthographic: *F*_(1,34)_ = 32.4, *p* < 0.001, *η* = 0.48; Phonological: *F*_(1,34)_ = 25.4, *p* < 0.001, *η* = 0.42). No other effects were significant (*p*s > 0.05; see Figure 2).

*The LRP analysis (400–600 ms)* showed a different pattern of results according to the deviancy type: Visual-orthographic deviants elicited a significant Deviancy * Group interaction, such that only dyslexic readers showed an LRP increase to visual deviants compared with standards; a similar modulation was not apparent in typical readers (*F*_(1,34)_ = 4.4, *p* < 0.05, *η* = 0.12). No other effects were significant for visual-orthographic deviants (*p*s > 0.05). There was significant main effect of Deviancy, such that phonological deviants produced larger LRPs (*F*_(1,34)_ = 20.16, *p* < 0.001, *η* = 0.37). No other effects were significant (*p*s > 0.05; see Figure 3).

## Experiment 2: Method

### Participants

The same participants who took part in Experiment 1 took part in Experiment 2.

### Stimuli, Design and Procedure

The design and procedure were identical to Experiment 1, but the stimuli in each trial comprised a geometric shape (again subtending a visual angle of 1 degree) paired with an auditory tone (low or high pitch) synchronized to the onset of the shape.

*Target* trials (*n* = 72 in total)—which required a button press response—always comprised a circle presented with a high-pitched (1500 Hz) tone.

**Figure 4:**
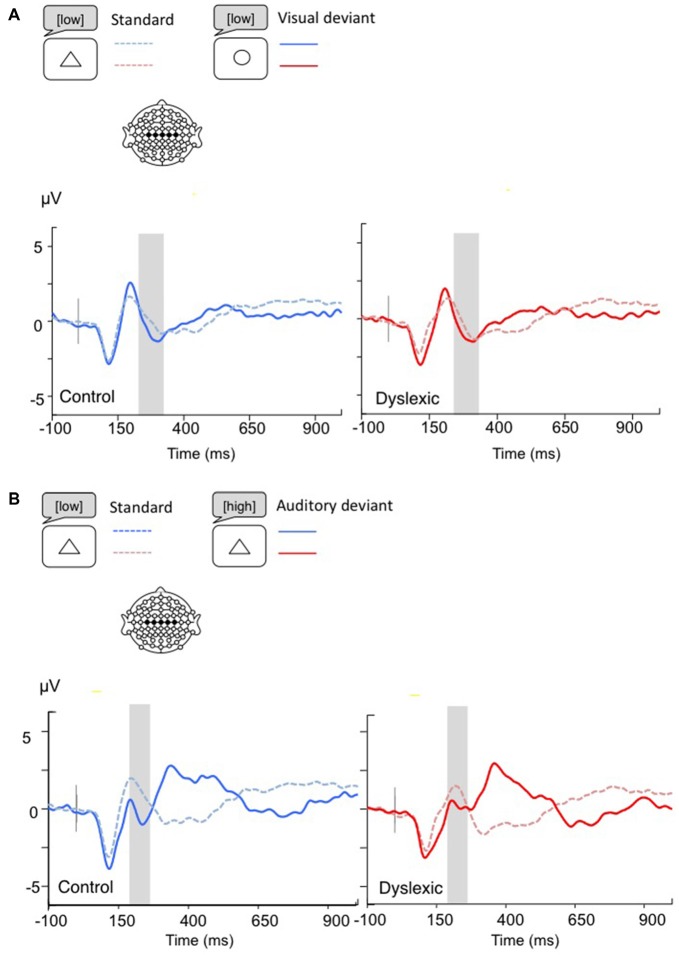
**Linear derivations over the electrodes where the MMN was maximal for typical and dyslexic readers’ responses in correct trials (Gray bar: analysis window). (A)** Visual deviants; **(B)** Auditory deviants.

**Figure 5:**
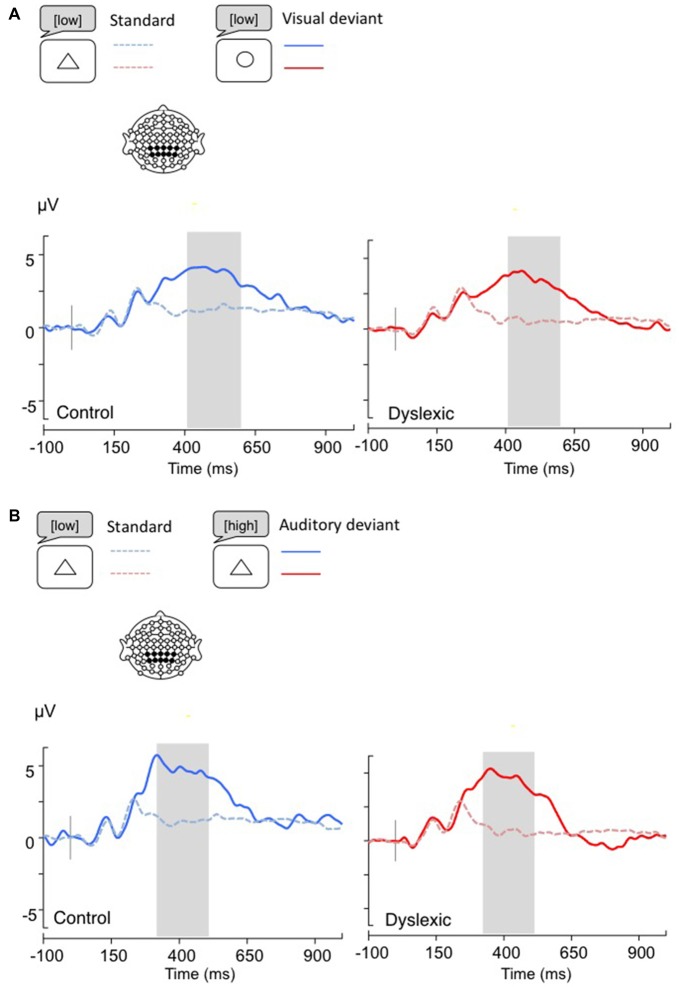
**Linear derivations over the electrodes where the P3b was maximal for typical and dyslexic readers’ responses in correct trials (Gray bar: analysis window). (A)** Visual deviants; **(B)** Auditory deviants.

**Figure 6:**
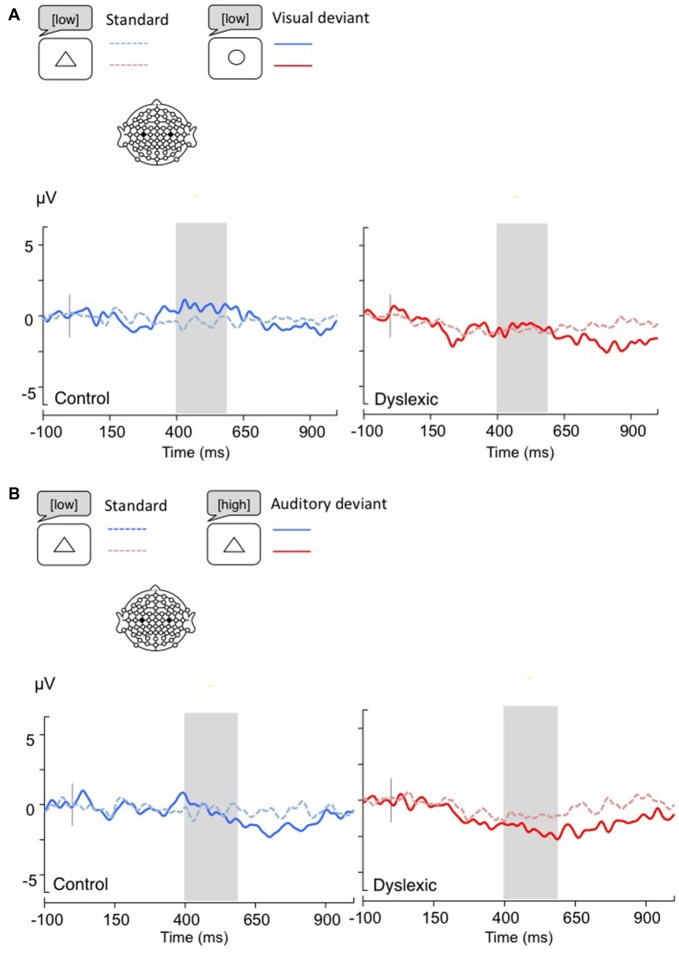
**LRPs, calculated by subtracting C4 amplitudes from C3.** Gray bar: analysis window. **(A)** Visual deviants; **(B)** Auditory deviants.

*Deviant* trials (*n* = 72 in total)—which did not require a button press response—comprised either a circle presented with a low-pitched tone (visual deviant; *n* = 36) or a square presented with a high-pitched tone (auditory deviant; *n* = 36; see Figures 4–6).

*Standard* trials (*n* = 360 in total) comprised one of 36 shapes paired with a low-pitched (900 Hz) tone. Note that no distinction was made in Experiment 2 between general and super standards, owing to the circumscribed number and nature of the target and deviant stimuli. In general, shapes were visually uncomplicated and the same low tone (also presented in visually deviant trials) was presented throughout.

### ERP Data Analysis

Mean ERP amplitudes were calculated and analyzed in the same way as in Experiment 1. Visual inspection of the grand average waveforms revealed that N2 and P3 latencies were shorter than in Experiment 1. This may be due to the less complex stimuli and the task. Therefore, different time-windows were selected for analysis. In addition, the MMN modulation was maximal over slightly more frontal regions than in Experiment 1, and the electrodes F3, F4, FC1, FC2, FCZ, FZ, were selected for analysis. The MMN moreover peaked in a slightly later time window for visual deviants, compared with auditory deviants. Peak detection and mean amplitudes of the P3b were conducted in the time-window 280–480 ms post stimulus onset over the electrodes CP1, CP2, CPz, CP3, CP4, P1, P2, Pz, P3, P4 (same as Experiment 1). Manual response preparation was measured via the LRP.

Mean MMN, P3b, and LRP amplitudes were subjected to mixed model ANOVAs, comprising the within-subject factor Deviancy (standard shape vs. deviant visual) and the between-subjects factor Group (typical vs. dyslexic readers). Only the standard trials immediately preceding the target/deviant trials were included in the analyses. ERP analyses were conducted only on correctly judged deviants (i.e., the participant did not execute a button press response). “Correct” trials were again excluded from all analyses.

### Results

#### Behavioral Results

Both reading groups showed the same degree of accuracy in detecting correct trials (Typical *M* = 98%; Dyslexic *M* = 98%; *b* = 1.12, *z* = 1.61, *p* = 0.11), but dyslexic readers responded more quickly on average (Typical *M* = 541 ms; Dyslexic *M* = 425; *b* = −116, *t* = 6.66, *p* < 0.001). In response to incongruent but similar pairs (“deviant” trials), typical and dyslexic readers committed a similar number of errors—erroneously committing a button press response—in visual trials (Typical *M* = 98%; Dyslexic *M* = 98%; *b* = 0.39, *z* = 0.84, *p* = 0.39) and auditory trials (Typical *M* = 99%; Dyslexic *M* = 99%; *b* = −0.25, *z* = −0.42, *p* = 0.67).

#### ERP Results

A series of *t*-tests showed no significant group differences on the number of trials per condition included in the analyses (Standards: Typical readers *M* = 106, *SD* = 16; Dyslexic readers *M* = 109, *SD* = 9; *t* = 0.54; Visual deviants: Typical readers *M* = 36, *SD* = 3; Dyslexic readers *M* = 36, *SD* = 2; *t* = 0.91; Sound deviants: Typical readers *M* = 34, *SD* = 5; Dyslexic readers *M* = 35, *SD* = 4; *t* = 0.80).

*The MMN analysis (Visual: 230–330 ms; Auditory: 200–270 ms)* showed a significant main effect of Shape deviancy, indicating an increased N2 for both deviants compared with standard trials, (Visual: *F*_(1,34)_ = 10.36, *p* < 0.01, *η* = 0.23; Auditory: *F*_(1,34)_ = 5.83, *p* < 0.05, *η* = 0.15). No other effects were significant (all *p*s > 0.05; see Figure 4).

*The P3b analysis (Visual: 400–600 ms; Auditory: 300–500 ms)* showed a significant effect of Shape deviancy, indicating an increase in the P3b wave for deviant trials (Visual: *F*_(1,34)_ = 14.8, *p* < 0.001, *η* = 0.30; Auditory: *F*_(1,34)_ = 7.2, *p* < 0.05, *η* = 0.17). No other effects were significant (all *p*s > 0.05; see Figure 5).

*The LRP analysis (400–600 ms)* showed no significant results (all *p*s > 0.05; see Figure 6).

## Discussion

The current study examined whether the core deficit of visual-phonological integration in developmental dyslexia arises from impaired visual-orthographic or phonological processing.

Typical readers detected both visual and phonological deviants as early as the N2 window, with deviants eliciting larger PMN amplitudes than standards. A larger P3b modulation was also elicited in response to deviants, whilst an increased negativity of the LRP was found specifically for *phonological* but not visuo-orthographic deviants. Dyslexic readers were just as accurate as typical readers in their responses to *correct* letter pairs, but the behavioral data also indicated a trend for a speed-accuracy trade-off: members of the dyslexic group made faster responses to correct trials, but made more errors to visual-orthographic deviants. Their electrophysiological responses also indicated abnormal letter processing since they showed no evidence of sensitivity to the deviancy manipulation (neither visual nor phonological) in the PMN time-window. However, dyslexic and typical readers showed similar P3b modulations. The LRP analysis revealed increased negativity in response to phonological deviants commensurate with typical readers’ responses, but crucially, the dyslexic group also showed increased negativity in response to visual-orthographic deviants: an effect that was completely absent in typical readers. In Experiment 2, pairs of previously unlearned visual-auditory items elicited P3b effects that indicated detection of visual and auditory deviants relative to baseline standards, analogous to the effect found in Experiment 1. No other effects emerged in the MMN or the LRP analyses, and crucially, both ERP and behavioral data indicated no reading group differences on this task.

Recent studies using a range of methodologies have established that dyslexia involves weaker links between visual-orthorgraphic and phonological representations. Behavioral PAL studies have shown that the ability to learn novel visual-verbal correspondences significantly predicts word reading and discriminates reading ability (Vellutino et al., 1995; Wimmer et al., 1998; Messbauer and de Jong, 2003; Hulme et al., 2007; Warmington and Hulme, 2012). Consistent with this result, imaging studies have shown reduced levels of activitiy in the superior temporal gyrus, planum temporale, and superior temporal sulcus in children with poor reading ability (c.f., Blau et al., 2010), and evidence from ERPs has revealed impaired automaticity in the initial stages of letter processing (Froyen et al., 2011).

First, our findings corroborate previous evidence that dyslexic readers do not automatically integrate letters with auditory letter names during the early stages of letter processing. We moreover show that this effect is amodal: dyslexic readers are relatively insensitive to visual-orthographic *and* phonological similarity during the early stages of letter processing. Downstream, dyslexic readers’ explicit recognition of letters—indicated by modulation of P3b amplitudes—show normal sensitivity to the visual-orthographic and phonological characteristics of letters. However, when required to select one of these representations for output, visual-orthographic similarity becomes a problem, leading to uncertainty in preparing a motor response, and an increase in error rate. Our findings reveal a shift in visual-orthographic and phonological sensitivity as a function of reading ability: typical readers are highly tuned to visual-orthographic and phonological letter properties from an early processing stage, whereas dyslexic readers activate this information later; potentially precluding efficient selection of the letter’s identity from competing alternatives during the output/decision stage (c.f., Rispens, 2004; Schulte-Körne et al., 2004; Jones et al., 2008, 2013a; Savill and Thierry, 2011, for concordant evidence of an ouput deficit in dyslexia). Note that the current paradigm did not require an overt vocal response, further supporting the hypothesis of a fault at the selection stage (Fawcett and Nicolson, 2002).

Our findings are consistent with an account of dyslexia specifying an impairment in phonological recoding, that is, the ability to translate a printed letter or letter string into its spoken form, enabling development of an autonomous orthographic lexicon (Jorm and Share, 1983; Share, 1995; see also Badian, 2001; Cunningham et al., 2001). On this account, the integrity of orthographic representations is dependent on feedback from corresponding phonological representations, with fluency developing as a function of repeated exposures to orthographic and phonological forms (Ehri and Saltmarsh, 1995; Ehri, 2005a,b). Compromised neural links in the formation of visual-orthographic and phonological bindings in dyslexia would lead to relative underspecification of orthographic representations, despite repeated exposure during reading development.

We thus suggest that adult dyslexic readers fail to form precise, automatic visual-phonological mappings, with consequences for the ability to verify the *visual-orthographic* characteristics of print for stimulus selection and output, consistent with other recent findings in the field of RAN (e.g., Jones et al., 2013a, 2015). Two further points should be considered here. First, we find no evidence in the current study to support a primary phonological deficit as a possible cause of the putative recoding impairment. And second, any underspecification of orthographic representations in these adult, high-functioning dyslexic readers cannot be severe, since our data indicated normal sensitivity to visual-orthographic characteristics in the context of letter recognition.

To conclude, this study used a letter-matching task to examine the integrity of visual-orthogaphic and phonological links in developmental dyslexia. Our findings show that adult, high functioning dyslexic readers can develop orthographic representations that are sufficiently specified to fully activate likely candidates, based on the input. However, their apparent failure to automatize visual-phonological connections impairs their ability to select a single representation for output, a process that relies not only on highly specified orthographic representations, but also robust visual-to-phonological mapping.

## Author Contributions

MWJ, J-RK, and GT designed the research; MWJ and J-RK performed the research and analyzed the data; MWJ, J-RK, and GT wrote the article.

## Conflict of Interest Statement

The authors declare that the research was conducted in the absence of any commercial or financial relationships that could be construed as a potential conflict of interest.

## Footnotes

^1^The effects found in this study are rather more central than the “typical” frontocentral PMN found previously (c.f., Connolly et al., 1992; Connolly and Phillips, 1994), and show a later onset (320 ms post stimulus, compared with the more typical 275 ms). Slight deviations in topography and onset are likely due to task differences: The current task involved visual-auditory letter integration whereas typical PMN studies involve violations of phonological expectancy during sentence reading.
